# High-density mapping of the average complex interval helps localizing atrial fibrillation drivers and predicts catheter ablation outcomes

**DOI:** 10.3389/fcvm.2023.1145894

**Published:** 2023-08-17

**Authors:** Fabien Squara, Didier Scarlatti, Sok-Sithikun Bun, Pamela Moceri, Emile Ferrari, Olivier Meste, Vicente Zarzoso

**Affiliations:** ^1^ Cardiology Department, Pasteur Hospital, Université Côte d’Azur, Nice, France; ^2^ I3S Laboratory, Université Côte d’Azur, CNRS, Sophia Antipolis, France

**Keywords:** atrial fibrillation, ECG, ablation, dominant frequency, independent component analysis, average complex interval, spatiotemporal dispersion, drivers

## Abstract

**Background:**

Persistent Atrial Fibrillation (PersAF) electrogram-based ablation is complex, and appropriate identification of atrial substrate is critical. Little is known regarding the value of the Average Complex Interval (ACI) feature for PersAF ablation.

**Objective:**

Using the evolution of AF complexity by sequentially computing AF dominant frequency (DF) along the ablation procedure, we sought to evaluate the value of ACI for discriminating active drivers (AD) from bystander zones (BZ), for predicting AF termination during ablation, and for predicting AF recurrence during follow-up.

**Methods:**

We included PersAF patients undergoing radiofrequency catheter ablation by pulmonary vein isolation and ablation of atrial substrate identified by Spatiotemporal Dispersion or Complex Fractionated Atrial Electrograms (>70% of recording). Operators were blinded to ACI measurement which was sought for each documented atrial substrate area. AF DF was measured by Independent Component Analysis on 1-minute 12-lead ECGs at baseline and after ablation of each atrial zone. AD were differentiated from BZ either by a significant decrease in DF (>10%), or by AF termination. Arrhythmia recurrence was monitored during follow-up.

**Results:**

We analyzed 159 atrial areas (129 treated by radiofrequency during AF) in 29 patients. ACI was shorter in AD than BZ (76.4 ± 13.6 vs. 86.6 ± 20.3 ms; *p* = 0.0055), and mean ACI of all substrate zones was shorter in patients for whom radiofrequency failed to terminate AF [71.3 (67.5–77.8) vs. 82.4 (74.4–98.5) ms; *p* = 0.0126]. ACI predicted AD [AUC 0.728 (0.629–0.826)]. An ACI < 70 ms was specific for predicting AD (Sp 0.831, Se 0.526), whereas areas with an ACI > 100 ms had almost no chances of being active in AF maintenance. AF recurrence was associated with more ACI zones with identical shortest value [3.5 (3–4) vs. 1 (0–1) zones; *p* = 0.021]. In multivariate analysis, ACI < 70 ms predicted AD [OR = 4.02 (1.49–10.84), *p* = 0.006] and mean ACI > 75 ms predicted AF termination [OR = 9.94 (1.14–86.7), *p* = 0.038].

**Conclusion:**

ACI helps in identifying AF drivers, and is correlated with AF termination and AF recurrence during follow-up. It can help in establishing an ablation plan, by prioritizing ablation from the shortest to the longest ACI zone.

## Introduction

Catheter radiofrequency ablation is the therapy of choice in symptomatic drug-refractory Persistent Atrial Fibrillation (PersAF). Empirical pulmonary vein isolation (PVI) remains the cornerstone of the ablation strategy (1). However, many additional substrate-based ablation strategies have been developed for improving ablation results, but the most appropriate target for PersAF ablation is still under debate.

As a potential target for PersAF ablation, Complex Fractionated Atrial Electrograms (CFAE) ablation has been described with variable success (2–4). One major issue when performing CFAE ablation is the difficulty in discriminating “active” – i.e., participating in AF maintenance – from “passive” fractionated zones. Since fractionated electrogram (EGM) distribution is variable and depends on direction and rate of activation of the myocardium, a significant proportion of CFAE may be functional in nature (5, 6). Several previous studies focused on the characteristics of CFAE that would predict favorable response to ablation, and highlighted the temporal gradient of activation between mapping electrodes and the percentage of continuous electrical activity as predictors of active CFAE zones (6–8). This has led to the advent of ablation guided by Spatiotemporal Electrogram Dispersion (STD) with promising preliminary results (9). However, despite these improvements in AF substrate understanding, the discrimination between active and passive atrial zones remains largely suboptimal (10). As such, new tools might help the electrophysiologist to target the most desirable atrial substrate zones for successful ablation.

The Average Complex Interval (ACI) is a feature embedded in the Carto system (Biosense Webster Inc., Irvine, CA, USA), allowing for an automated assessment of the average interval between EGM deflections at each recorded atrial point. This reflects the degree of complexity of the local atrial signal, and is related to the local AF cycle length. Using the contemporary high-density atrial mapping, the continuous ACI value might help the electrophysiologist to further identify the most appropriate substrate zones in addition to the binary detection of STD presence, since most complex electrograms appear to be localized to zones displaying the maximal local dominant frequency (DF) that are known to perpetuate AF (11).

In this study, we sought to determine the potential value of ACI for helping the electrophysiologist find the atrial zones that would be appropriate targets in the setting of PersAF radiofrequency ablation. To this end, we analyze the value of ACI for (1) discriminating AF Active Drivers (AD) from Bystander Zones (BZ) determined by the evolution of AF complexity by means of sequential DF computation on surface ECG during ablation, (2) predicting AF termination during radiofrequency ablation, and (3) predicting AF recurrence during follow-up.

## Methods

### Study population

Consecutive patients undergoing first time radiofrequency ablation for persistent or long-standing persistent symptomatic AF at Pasteur University Hospital, Nice, France, were included. Any patient with history of previous ablation within the left atrium was excluded. The study was approved by the institutional review board, and all patients gave written informed consent. Study data will be made available upon reasonable request to the corresponding author.

### Electroanatomical mapping and ablation procedure

Procedures were performed under general anesthesia. High-density electroanatomical mapping (>1,000 points) of the entire left atrium (LA) was performed in AF with the Carto system V6 or V7, using the Pentaray catheter (Biosense Webster Inc.) inserted transseptally via a non-steerable sheath (Fast-Cath SL0; St. Jude Medical, Minnetonka, MN, USA). Detailed mapping of electrical activation of the LA was performed, with live visual annotation by the electrophysiologist of zones of STD (defined by clusters of electrograms, either fractionated or non-fractionated, that displayed interelectrode time and space dispersion at a minimum of 3 adjacent bipoles such that activation spread over all the AF cycle length) (9) and of CFAE with continuous electrical activity during >70% of the recording, also using visual appreciation. At baseline, AF cycle length was measured in the left atrial appendage (LAA) by averaging 10 cycles. LA volume (ml) and LA surface (cm^2^) were assessed using high-density electroanatomical mapping with the Carto system, after excluding the inside portion of PVs and the mitral valve.

Point-by-point ablations were performed with endpoints of circumferential pulmonary veins (PV) disconnection, ablation at STD and CFAE sites, and block across the lines (if performed). Roof lines, anterior mitral lines or lateral mitral lines were performed in cases of presence of STD or CFAEs in the area. Each area was strictly ablated in a sequential manner, to allow assessment of the effect of each area ablation on AF DF. The order of the treatment of each documented atrial area was left to the physician's choice. LA areas were defined as: left PVs ostia, right PVs ostia, roof, anterior, septal, floor, lateral isthmus and LAA base.

High-power short duration irrigated radiofrequency was delivered using a power of 50 W and with an ablation index target of 350 Arbitrary Units (AU) on the posterior wall of the LA, and 420 AU elsewhere. If coronary sinus (CS) ablation was performed, radiofrequency power was limited to 25–30 W.

AF termination during ablation was defined as sinus rhythm resumption or its change to a stable atrial tachyarrhythmia. Nevertheless, this was not a procedural endpoint, and operators ended the procedures after PV isolation, complete ablation of the annotated STD and CFAE sites and, when appropriate, block across the lines. Associated atrial tachyarrhythmia ablation was performed. In cases without AF or atrial tachyarrhythmia termination at the end of the procedure, an electrical cardioversion was performed (200 J biphasic, repeated up to three times).

### Average complex interval measurement

Operators were blinded to the ACI measurements which were performed off-line after the procedure. The ACI is a feature embedded in the Carto System, which works by assessing the mean interval between automatically tagged EGM deflections recorded over a 2.5 s period. In order to be counted by the software, a deflection must meet amplitude and interval criteria that can be modified by the user. In this study, the amplitude window was set as 0.05 mV (noise level) to 2 mV (to avoid counting of far field ventricular EGMs from sites near the mitral annulus), the minimal interval between two deflections was 50 ms (atrial refractory period), and the maximal interval was ≥200 ms.

**Figure 1:**
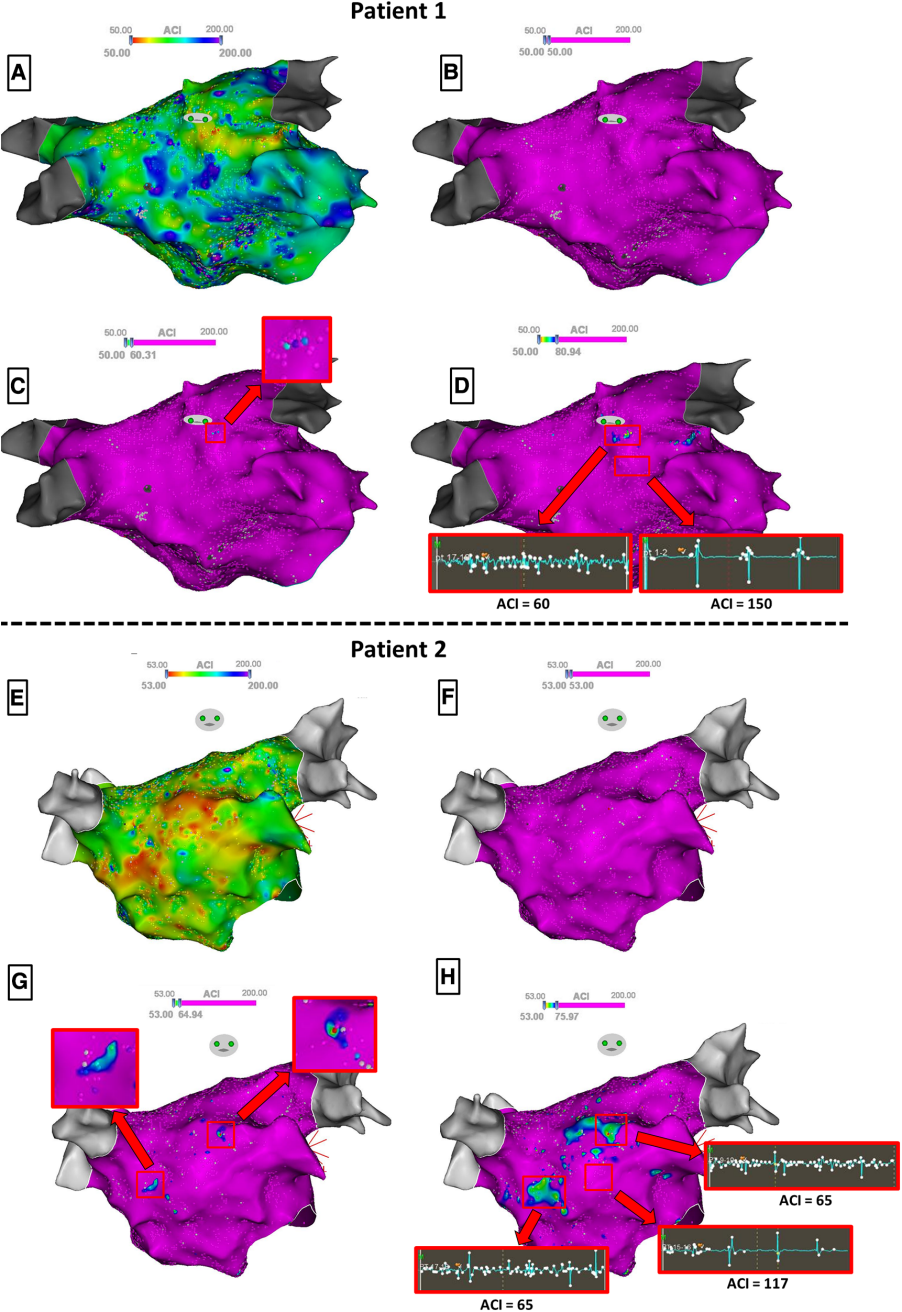
Steps for identifying the ACI value of atrial substrate zones in two patients. To ease visualization, manual annotations of STD/CFAE zones are hidden. Panels **A** and **E** display the raw ACI mapping, and panels **B** and **F** the ACI mapping with the highest ACI threshold set to the minimal value so that the entire map appears purple. Then, the cursor is progressively moved rightwards until atrial areas appear on the ACI map with at least three adjacent points displaying any color but purple (Panels **C** and **G**), defining the ACI of the zone. Note that in patient 1 (Panel **C**), only one zone flashes out whereas in patient 2 (Panel **G**), two zones appear simultaneously with an identical ACI value. When further moving rightwards the cursor (Panel **D** and **H**), the detected zones now spread around the shortest ACI values. Samples of the 2.5 s EGM recordings for ACI measurements are shown.

Then, the ACI mapping of the LA was displayed, and we measured the ACI value of each atrial area that was previously tagged as STD or CFAE during the procedure. To achieve this, the cursor of the highest ACI threshold was first set to the minimal value so that the entire map appears purple; then, it was progressively moved rightwards until each tagged atrial area appeared on the ACI map with at least three adjacent points displaying any color but purple (Figure 1, panels C and G). This minimal ACI value for three adjacent points of an atrial area to appear on the ACI map defined the ACI of the area. Temporal stability and validity of ACI measurements were confirmed by means of sequential mapping: presence of ≥3 adjacent points presenting identical shortest ACI value in each atrial area, and immediately surrounding points demonstrating a progressive increase of ACI value (Figure 1, panels D and H).

### Other assessed electrophysiological parameters

We also recorded the surface of each atrial substrate zone, the ratio between the surface of each atrial substrate zone and total LA surface, the ratio between the surface of each atrial substrate zone and the total surface of atrial substrate, the integration of the treated atrial zone into a complete line of block, the localization of each atrial substrate zone, and the order of the treated zone during the ablation procedure.

### Signal acquisition and processing

#### ECG signal acquisition

ECG signals were acquired on a digital electrophysiological recording system (Labsystem Pro EP, Boston Scientific, Marlborough, MA, USA), including 0.05–40 Hz band pass and 50 Hz notch filters. For every patient, a 1-minute standard 12-lead ECG in AF was recorded at a sampling rate of 1,000 Hz at the beginning of the procedure and after each LA area ablation.

#### AF dominant frequency assessment

AF DF is related to the refractory period of atrial myocardium cells, and thus to the degree of complexity of the disease and the probability of spontaneous cardioversion (12). We favored DF measure over sequential LAA cycle assessment because regional radiofrequency ablation in the vicinity of LAA can affect LAA cycle, and as such, it might not reliably reflect AF complexity evolution during the ablation procedure. In this work, we assessed DF at every step of the ablation procedure by means of Robust Independent Component Analysis in the frequency domain (RobustICA-f). This approach to atrial activity extraction relies on the observation that atrial activity and ventricular activity can be considered statistically independent phenomena during AF (13). Techniques for the separation of independent signals such as RobustICA-f can then be applied on the 12-lead ECG to search for the atrial activity source, thus allowing the reconstruction of atrial activity in all leads free from ventricular activity and other interference. Details on the technique have been given previously (14).

**Figure 2:**
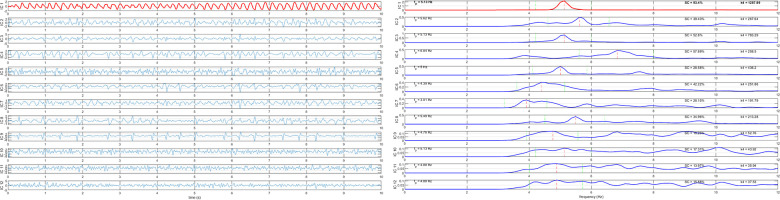
Signal processing for dominant frequency measurement using RobustICA-f. Left panel: separation of the independent signals acquired from the 12-lead ECG; to ease visualization, only 10 s of the recordings are shown. Right panel: frequency spectra of the signals shown in the left panel. The atrial activity source is identified as the extracted component with dominant frequency in the interval [3–9] Hz with the highest spectral concentration value. In both panels, the identified atrial activity source is displayed in red.

The atrial activity source is automatically selected as the extracted component with the highest spectral concentration among the sources with dominant peak in the interval [3–9] Hz, the typical AF frequency band. RobustICA-f was implemented using MATLAB, version 2020b (MathWorks). Figure 2 illustrates the signal processing for DF measurement using RobustICA-f.

### Discrimination between active drivers and bystander zones

AD were differentiated from BZ either by a significant decrease of the AF DF measured by RobustICA-f after ablation of the atrial area, or by AF termination during ablation of the area.

**Figure 3:**
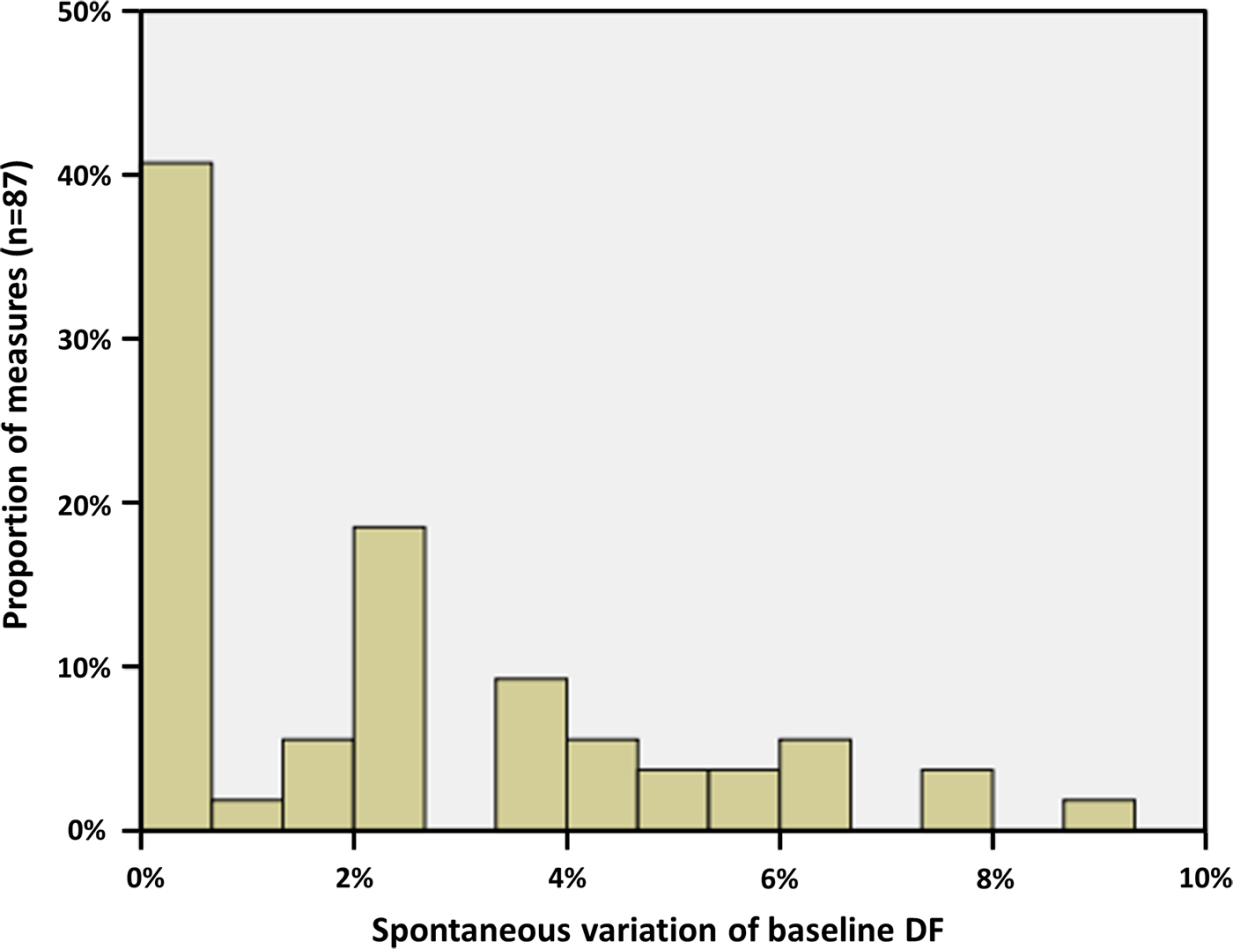
Spontaneous evolution of basal AF DF, measured under general anesthesia on three randomly taken ECG in every patient. The maximal spontaneous variation was 8.8%; thus, a cut-off value of 10% has been chosen for determining a significant evolution after ablation.

In order to establish a cut-off value from which a variation of the DF would be considered significant, we first performed three measurements of the basal DF in every patient, before any ablation. These measurements were obtained from three 1-minute ECGs taken randomly in a period of time of 30 min, under general anesthesia. Spontaneous variation of the baseline DF varied from 0% to a maximum of 8.8% (Figure 3). Accordingly, a variation of >10% was taken as a strong cut-off value to determine a significant decrease of the DF after ablation steps.

### Follow-up

A blanking period of 1 month was chosen in this study, since early arrhythmia recurrences at 1–3 months have proven to be predictive of late arrhythmia recurrences (15–17). After this 1-month blanking period, patients were followed for clinical and asymptomatic recurrences for at least 12 months. Follow-up was performed in a real-life setting, by regular visits to the treating cardiologist, with repeated ECG and 24-hour Holter monitoring in all cases (every 3 months during the first year after ablation; every 6 months afterwards). Supplementary documentation by ECG or Holter was sought in case of recurring symptoms suggestive of arrhythmia. Recurrence of sustained (>30 s) AF or flutter was recorded.

### Statistical analysis

Normal distribution of continuous variables was checked with the Kolmogorov–Smirnov test. Categorical variables were presented as percentages and continuous variables as mean and standard deviation in case of normal distribution, or median and confidence interval otherwise. Under data normality, groups were compared by a parametric unpaired or paired *t*-test as appropriate, whereas a non-parametric Mann–Whitney *U*-test was used when the variables did not show a normal distribution. Proportion analysis was based on the *χ*^2^ test. Predictive value of the ACI was assessed using the receiving operator characteristics (ROC) curve analysis. Multivariate analysis was made using logistic regression. Covariates were assessed initially in a univariate fashion, and those with a *p*–value of ≤ 0.1 were entered into the multivariate model. Statistical significance was defined by a *p*-value < 0.05.

## Results

A total of 159 atrial areas were documented as displaying STD or continuous CFAEs and were analyzed, in a study cohort of 29 patients (age 67.9 ± 9.2 years; 27.6% females). Amongst these atrial areas, 129 were treated by radiofrequency application during AF. The remaining 30 areas were either not ablated because AF termination occurred previously, or ablated in atrial tachycardia or in sinus rhythm; therefore, sequential analysis of AF DF was not performed for these areas. Clinical and procedural characteristics are given in Table 1. Mean AF duration was 21.7 ± 20.4 months, and 15/29 patients (51.7%) had a long-standing persistent AF (>12 months). AF cycle length measured in the LAA at baseline was 180.4 ± 24.3 ms. We performed a high resolution electroanatomical mapping of the LA substrate with 3,171 ± 1,864 points acquired with the Carto system. We documented 5.5 ± 1.5 (from 2 to 8) atrial zones of STD or continuous CFAEs per patient. The total surface of LA zones with STD or CFAEs was 21.3 ± 8.7 cm^2^ per patient, representing 8.9% ± 3.6% of the total LA surface.

**Table 1:** Clinical and procedural characteristics.

	*n* = 29
Age (y)	67.9 ± 9.2
Female gender (*n*; %)	8/29; 27.6%
AF duration (months)	21.7 ± 20.4
Left atrial volume (ml)	169.7 ± 37.1
AF cycle length in LAA (ms)	180.4 ± 24.3
Number of LA dispersion-fragmentation zones per patient (*n*)	5.5 ± 1.5
Total LA surface of dispersion-fragmentation zones (cm^2^)	21.3 ± 8.7
Total LA surface of dispersion-fragmentation zones/Total LA surface (%)	8.9 ± 3.6
Right pulmonary vein ostium dispersion-fragmentation zone (*n*; %)	26/29; 89.7%
Left pulmonary vein ostium dispersion-fragmentation zone (*n*; %)	26/29; 89.7%
Anterior dispersion-fragmentation zone (*n*; %)	21/29; 72.4%
Left atrial appendage ostium dispersion-fragmentation zone (*n*; %)	13/29; 44.8%
Roof dispersion-fragmentation zone (*n*; %)	19/29; 65.5%
Septal dispersion-fragmentation zones (*n*; %)	19/29; 65.5%
Floor dispersion-fragmentation zones (*n*; %)	6/29; 20.7%
Lateral mitral isthmus dispersion-fragmentation zones (*n*; %)	8/29; 27.6%
Basal AF dominant frequency (Hz)	5.31 ± 0.72
AF termination during radiofrequency ablation procedure (*n*; %)	19/29; 65.5%
Follow-up duration (months)	23.3 ± 9.8
AF recurrence (*n*; %)	2/29; 6.9%
Recurrence of any atrial arrhythmia (*n*; %)	13/29; 44.8%
Follow-up duration to any atrial arrhythmia recurrence (months)	4.0 (3.0–6.2)

### Characteristics of active drivers vs. bystander zones

Amongst the 129 atrial areas treated by radiofrequency application during AF, 41 (31.8%) led either to a significant decrease in AF DF (22 areas) or to AF termination (19 areas), and were classified as AD; the remaining 88 (68.2%) atrial areas were classified as BZ. Ablation of the AD areas not inducing AF termination led to a 14.0% ± 3.9% decrease in AF DF, vs. 4.4% ± 3.1% (*p* < 0.001) for BZ areas.

In univariate analyses, ACI parameters demonstrated significant differences between AD and BZ (Table 2). Mean ACI was significantly shorter in AD than in BZ (76.4 ± 13.6 vs. 86.6 ± 20.3 ms; *p* = 0.0055), and the ratio between ACI and AF cycle length in LAA was also smaller in AD than in BZ (41.8 ± 6.6 vs. 47.5% ± 10.0%; *p* = 0.0019).

**Table 2:** Active drivers vs. bystander zones (univariate analyses).

	Active driver (*n* = 41)	Bystander zone (*n* = 88)	*p*-value
Surface of the treated LA zone (cm^2^)	3.14 ± 2.11	4.31 ± 3.54	0.06
Surface of the treated LA zone/Total LA surface (%)	1.36 ± 0.8	1.8 ± 0.1	0.1204
Surface of the treated LA zone/Total surface of dispersion or fragmentation zones (%)	17.4 ± 10.7	19.4 ± 13.7	0.4425
Peri Pulmonary Vein zone (*n*; %)	19/41 (46.3%)	35/88 (39.8%)	0.481
Integration of the treated zone into a complete line of block (extra-pulmonary vein) (*n*; %)	6/22 (27.3%)	22/53 (41.5%)	0.246
ACI of the treated LA zone (ms)	76.4 ± 13.6	86.6 ± 20.3	0.0055*
ACI of the treated LA zone/AF cycle length in LAA (%)	41.8 ± 6.6	47.5 ± 10.0	0.0019*
Number of other zones with similar or lower ACI than the treated LA zone (*n*)	0.97 ± 1.24	2.12 ± 1.48	0.0001*
Order of the treated atrial zone during the ablation procedure	3.48 ± 2.12	2.93 ± 1.57	0.1

*denotes statistical significance (*p* < 0.05).

Also, importantly, the ACI value of all other zones than the assessed one had an influence on AD vs. BZ discrimination, and more specifically the number of other atrial zones having a shorter or an identical ACI value. Indeed, the number of other zones with shorter or similar (± 5 ms) ACI value was lower in zones determined as AD than in zones determined as BZ (0.97 ± 1.24 vs. 2.12 ± 1.48 zones; *p* = 0.0001). In other terms, the ablation of an atrial zone was less likely to induce AF DF decrease or AF termination–and thus be classified as AD–when several other atrial zones demonstrated a shorter or similar ACI value, highlighting the global interest of this parameter.

The other studied parameters did not demonstrate a significant difference in univariate analysis between AD and BZ: surface of the treated atrial zone (*p* = 0.06), ratio between the surface of the treated atrial zone and the total LA surface (*p* = 0.1204), ratio between the surface of the treated atrial zone and the total surface of STD or CFAEs (*p* = 0.4425), peripulmonary vein localization (*p* = 0.481), integration of the treated zone into a complete line of block (*p* = 0.246), and order of the treated zone during the ablation procedure (*p* = 0.1).

ACI predicted AD with an area under the ROC curve of 0.728 (0.629–0.826). The best documented threshold was an ACI < 70 ms for predicting AD, with a specificity of 0.83 but a low sensitivity (0.53). Conversely, an ACI value <100 ms was very sensitive (0.98) for predicting AD but with a specificity of 0.33. In other terms, atrial areas having an ACI > 100 ms had very little chances of being active in AF maintenance, while areas with an ACI value <70 ms were likely to reduce AF DF or to terminate AF during ablation.

In multivariate analysis (Table 3), AD was predicted by an ACI of the zone <70 ms [OR = 4.02 (1.49–10.84); *p* = 0.006], and by a number ≤2 of other atrial zones with similar or lower ACI than the considered zone [OR = 4.32 (1.33–14.05); *p* = 0.015]. The order of the treated atrial zone along ablation procedure had no influence to predict AD vs. BZ (OR =  1.06; *p* = 0.652). Finally, a smaller surface of the atrial substrate zone was associated with AD [OR = 0.76 (0.62–0.95); *p* = 0.009].

**Table 3:** Predictors of active drivers in multivariate analysis.

Predictor of Active Driver during ablation	Odds ratio	95% CI	*P* (Multivariate)
ACI of the treated atrial zone <70 ms	4.02	1.49–10.84	0.006*
Number of other atrial zones with similar or lower ACI than the treated zone ≤2	4.32	1.33–14.05	0.015*
Order of the treated atrial zone during the ablation procedure	1.06	0.82–1.37	0.652
Surface of the treated LA zone (cm^2^)	0.76	0.62–0.95	0.009*

*denotes statistical significance (*p* < 0.05).

### Predictors of AF termination with radiofrequency ablation

AF termination during radiofrequency was obtained in 19/29 patients (65.5%), the remaining 10 patients underwent electrical cardioversion at the end of the procedure.

In univariate analysis (Table 4), mean ACI of all documented atrial STD/CFAE zones was longer in patients for whom AF termination was reached per ablation as compared to those who underwent electrical cardioversion at the end of the procedure [82.4 (74.4–98.5) vs. 71.3 (67.5–77.8) ms; *p* = 0.0126], and the number of atrial zones having a similar (± 5 ms) ACI than the shortest ACI zone was smaller [0 (0–1) vs. 2 (0–4) zones; *p* = 0.0141]. Also, there was a higher gradient between ACI values of all STD/CFAE zones in patients for whom AF termination was reached per ablation; this translated into a greater mean difference between the fastest ACI zone and other STD/CFAE zones [15.2 (11.6–20.9) vs. 7.6 (3.9–13.5) ms; *p* = 0.0032].

**Table 4:** Af termination vs. No AF termination (univariate analyses).

	AF termination (*n* = 19)	No AF termination (*n* = 10)	*p*-value
AF duration (months)	9 (6–25.8)	30 (9.23–48)	0.0334*
Left atrial volume (ml)	143 (138–176)	193 (149–230)	0.0181*
AF cycle length in left atrial appendage (ms)	187 (163–197)	177 (160–188)	0.3137
Number of left atrial zones with dispersion-fragmentation	6 (5–7)	5 (3.3–6.7)	0.3233
Total left atrial surface with dispersion-fragmentation (cm^2^)	20.6 (15.8–27.2)	21.3 (11.5–30.4)	0.8291
AF dominant frequency in ECG (Hz)	5.37 (5.13–6.1)	6.1 (5.6–6.6)	0.042*
Mean ACI of all dispersion-fragmentation zones (ms)	82.4 (74.4–98.5)	71.3 (67.5–77.8)	0.0126*
ACI of the fastest dispersion-fragmentation zone (ms)	71.5 (62.3–80.0)	66.7 (63.9–73.6)	0.5328
Number of atrial zones with similar ACI than the fastest dispersion-fragmentation zone	0 (0–1)	2 (0–4)	0.0141*
Mean difference between fastest ACI zone and other dispersion-fragmentation zones (ms)	15.2 (11.6–20.9)	7.6 (3.9–13.5)	0.0032*

*denotes statistical significance (*p* < 0.05).

Additionally, baseline DF was lower in patients for whom AF termination was obtained [5.37 (5.13–6.1) vs. 6.1 (5.6–6.6) Hz; *p* = 0.042], as well as AF duration [9 (6–25.8) vs. 30 (9.23–48) months; *p* = 0.021] and LA volume [143 (138–176) vs. 193 (149–230) ml; *p* = 0.014].

The mean ACI value of all documented atrial zones predicted AF termination with an area under the ROC curve of 0.789 (0.619–0.959). The best point on the ROC curve for predicting AF termination was a mean ACI > 75 ms (Se 0.78, Sp 0.80).

In multivariate analysis (Table 5), a mean ACI value >75 ms predicted AF termination with OR = 9.94 (1.14–86.7), *p* = 0.038.

**Table 5:** Predictors of AF termination in multivariate analysis.

Predictor of AF termination during ablation procedure	Odds ratio	95% CI	*P* (Multivariate)
AF duration ≤12 months	5.04	0.59–42.9	0.139
Left atrial volume <175 ml	2.47	0.35–17.6	0.365
AF dominant frequency in ECG < 6 Hz	2.62	0.37–18.5	0.335
Mean ACI of all fragmentation-dispersion zones >75 ms	9.94	1.14–86.7	0.038*

*denotes statistical significance (*p* < 0.05).

### Arrhythmia recurrence during follow-up

After a follow-up of 23.3 ± 9.8 months, 2/29 patients (6.9%) had AF recurrence and 11/29 patients (37.9%) demonstrated left atrial flutter; 16/29 (55.2%) remained free from any arrhythmia after a single ablation procedure (Table 6).

**Table 6:** Characteristics in patients without AF recurrence during follow-up, compared to recurring patients.

	No AF recurrence (*n* = 27)	AF recurrence (*n* = 2)	*p-*value
AF duration (months)	12 (6–27.3)	72 (48–96)	0.0221*
Left atrial volume (ml)	165 (141–177)	238 (226–251)	0.0252*
AF cycle length in left atrial appendage (ms)	185 (169.5–190)	163.5 (157–170)	0.2646
Number of left atrial zones with dispersion-fragmentation	5 (5–6)	7 (6–7)	0.2514
Total left atrial surface with dispersion-fragmentation (cm^2^)	21.3 (16.1–26.7)	28.5 (17.8–39.2)	0.3722
AF termination during ablation procedure (*n*; %)	19/27 (70%)	0/2 (0%)	0.043*
AF dominant frequency in ECG (Hz)	5.62 (5.24–6.09)	6.53 (6.47–6.59)	0.0371*
Mean ACI of all dispersion-fragmentation zones (ms)	75.7 (71.8–91.5)	68.1 (67.2–69)	0.0901
ACI of the fastest dispersion-fragmentation zone (ms)	68.3 (65.0–75.8)	64.2 63.5–65.0)	0.3261
Number of atrial zones with similar ACI than the fastest dispersion-fragmentation zone	1 (0–1)	4 (3–4)	0.021*
Mean difference between fastest ACI zone and other dispersion-fragmentation zones (ms)	13.9 (10.9–16.7)	5.9 (4.7–7.2)	0.061

*denotes statistical significance (*p* < 0.05).

Patients with recurring AF had a longer AF duration before ablation [72 (48–96) vs. 12 (6–27.3) months; *p* = 0.0221], a larger LA [238 (226–251) vs. 165 (141–177) ml; *p* = 0.0252], a higher DF [6.53 (6.47–6.59) vs. 5.62 (5.24–6.09) Hz; *p* = 0.0371], and a greater number of atrial zones with similar ACI than the fastest ACI zone [4 (3–4) vs. 1 (0–1) zones; *p* = 0.021].

No studied parameter was different in patients recurring with any type of arrhythmia (AF or atrial flutter) than in non-recurring patients.

## Discussion

### Main results

In this study, we aimed at determining readily measurable electrophysiological characteristics that would help the cardiologist to find the most appropriate substrate zones in the setting of PersAF radiofrequency ablation. Several different levels of response to ablation were assessed: the impact of each individual atrial area ablation on AF complexity, the ability to terminate AF, and the recurrence of AF during follow-up. Our results suggest that ACI parameters are valuable in identifying the most appropriate atrial targets when used on top of visual STD or continuous CFAE determination, although the long-term clinical benefit of ablation of these atrial areas remains to be proven.

Our main results are as follows: (1) An ACI value <70 ms is specific for discriminating active drivers from bystander zones; (2) atrial zones with an ACI >100 ms are very likely bystanders; (3) ablation of an atrial zone has little chances of inducing a reduction in AF complexity if several other atrial zones having a shorter ACI value have not been previously ablated; (4) AF termination is more likely to occur when the average ACI value of all substrate zones is >75 ms; (5) AF termination is more likely to occur when the ACI gradient between all substrate zones is high; and (6) the risk of AF recurrence after ablation seems higher when several atrial zones have ACI values similar to the shortest one.

### Active and passive CFAE zones

CFAE ablation has been described long ago by Nademanee et al. and has led to variable success rates (2–4). One important issue is that electrogram fractionation is dependent on the direction and the rate of activation (5, 6), and therefore, a significant proportion of CFAE have a functional nature and are unrelated to AF maintenance. In a study using magnetic resonance imaging (MRI) with delayed enhancement for identifying atrial fibrosis, Jadidi et al. (18) found that nearly half of the areas displaying CFAE did not demonstrate any scar in MRI, while most of the remaining CFAE areas arose in patchy delayed enhancement zones. Discrimination between passive and active CFAE zones – participating in AF maintenance and localized in the vicinity of atrial tissue fibrosis which is known to harbor AF drivers (19–21) – is thus critical for appropriate AF substrate targeting.

Some electrophysiological characteristics such as the temporal gradient of activation between mapping electrodes and the percentage of continuous electrical activity have been previously described as predictors of active CFAE zones (6–8). In the present study, we attempted to add more precision to the discrimination between active and passive zones by performing a high resolution mapping of the ACI on top of visual annotation of continuous CFAE (>70% of the sample) and STD areas. Our favorable results demonstrate the interest of ACI mapping in the setting of PersAF ablation. However, attention should not only be paid to the ACI of a specific atrial zone, but rather on a wider scale to the ACI of every annotated atrial substrate zones, in order to prioritize the ablation plan. Indeed, AF has a complex physiopathology and numerous drivers may coexist at the same time (22). As a consequence, AF complexity might visibly remain unchanged even after ablation of a driver, due to remaining drivers that might be faster and perpetuate AF. The interplay between all these coexisting drivers is critical to acknowledge and is a plausible explanation for the lack of impact on AF complexity of ablation of atrial zones when remaining non-ablated areas display a shorter ACI.

The ACI value reflects the degree of fragmentation of the signal, and is correlated with the local AF cycle length. In a previous study (11), atrial electrograms demonstrated the highest degree of fractionation at the core or in immediate proximity of zones displaying the maximal dominant frequency, which are known maintainers of AF (23–25). This is the most likely physiopathological explanation for our favorable results using ACI: shortest ACI zones permit localization of the fastest local dominant frequency zones, explaining the positive impact of their ablation on AF complexity, especially when there are no other shorter ACI zones maintaining AF. Additionally, Lin et al. (11) also found that a higher gradient of dominant frequency in LA had a better response to catheter ablation. These findings are comparable to ours using ACI, where a higher gradient of ACI values in LA was found in patients for whom AF termination was reached per-ablation.

Several previous studies have confirmed that AF DF was a marker of procedural success (26, 27), however, studies specifically targeting highest DF zones for AF ablation have led to disappointing results (28, 29). Possible explanations for this apparent paradox are the low density of electroanatomical mapping (≈300 points per map) used in these study procedures performed more than a decade ago, and the variability and reproducibility of intracardiac DF assessment due to changes in amplitude and morphology of the EGMs which can impair the reliability of the measurement using Fourier analysis, especially when sample duration is short and mapping density is low (30). As current 3D-mapping technologies now allow very high density mapping but do not embed DF measurement software, ACI mapping might be an interesting alternative for a more targeted ablation approach.

Finally, we found in the present study that small LA zones displaying STD or CFAE were more likely to be active in AF maintenance than large ones. Non-contact mapping of AF sources and rotors (22, 31) revealed that the size of the drivers usually approximated 2 cm^2^. Hence, atrial CFAE or STD zones largely exceeding 2 cm^2^ might be considered as likely functional, i.e., passive areas.

Nevertheless, the optimal strategy for persistent AF ablation is still a much debated issue, and it is unclear if electrogram-based ablation after PV isolation is superior to PV isolation alone. Typically after STD ablation, AF recurrence is low but at the price of frequent recurrent atrial flutter/tachycardia (9). In our study, only 6.9% of the patients presented AF recurrence despite a mean AF duration before ablation of 21.7 months, with most patients having long-standing persistent AF; however, 37.9% of the patients recurred with atrial flutter.

### Clinical implications

The results of our study suggest that when EGM-based substrate ablation is sought during PersAF ablation, a high-density ACI mapping may be useful on top of visual identification of STD and continuous CFAE zones for establishing an ablation plan. One should prioritize ablation from the shortest to the longest ACI zone with a specific focus on zones having an ACI <70 ms, since shortest ACI indicate a close proximity to highest local DF zones and are more likely to be active in AF maintenance. However, areas having an ACI value >100 ms are likely functional (i.e., passive), and should therefore not be ablated.

Additionally, the presence of multiple short ACI zones–inducing a low mean ACI value and a low gradient between ACI zones is predictive of AF termination failure and AF recurrence.

Prospective randomized studies are warranted to evaluate whether the help of the ACI for determining the most appropriate zones leads to better ablation outcomes.

### Limitations

Our database is relatively small, with 29 patients included. The assessment of AF DF was carried out by means of atrial source separation taking into account all ECG leads simultaneously, and it is unknown whether sequential single lead analyses using ventricular cancellation based on average beat subtraction followed by the Fourier Transform method would have given comparable results.

Since ACI is measured on 2.5 s EGM samples, it is very important to perform a high-resolution mapping for replicating ACI assessment; sequential acquisitions will thus ensure temporal stability and validity of ACI measurements at every atrial zone, with shortest ACI values surrounded by points demonstrating a progressive increase of ACI. Also, ACI value might be flawed by artifacts or poor contact of the mapping electrode. Restrictive settings of the tissue proximity indicator (TPI) of the Carto system can help in decreasing points with poor contact; nonetheless, careful review of all shortest ACI zones is critical.

## Conclusion

ACI helps in identifying AF drivers, is correlated with AF termination and seems to be correlated with AF recurrence during follow-up. The ACI mapping may be useful on top of binary visual identification of STD and continuous CFAE zones for establishing an ablation plan, in order to prioritize ablation from the shortest to the longest ACI zone.

## Data Availability

The raw data supporting the conclusions of this article will be made available by the authors, upon reasonable request.

## References

[1] Hindricks G Potpara T Dagres N Arbelo E Bax JJ Blomström-Lundqvist C 2020 Esc guidelines for the diagnosis and management of atrial fibrillation developed in collaboration with the European association for cardio-thoracic surgery (eacts): the task force for the diagnosis and management of atrial fibrillation of the European society of cardiology (esc) developed with the special contribution of the European heart rhythm association (ehra) of the esc. Eur Heart J. (2021) 42:373–498. 10.1093/eurheartj/ehaa612 32860505

[2] Nademanee K McKenzie J Kosar E Schwab M Sunsaneewitayakul B Vasavakul T A new approach for catheter ablation of atrial fibrillation: mapping of the electrophysiologic substrate. J Am Coll Cardiol. (2004) 43:2044–53. 10.1016/j.jacc.2003.12.054 15172410

[3] Verma A Mantovan R Macle L De Martino G Chen J Morillo CA Substrate and trigger ablation for reduction of atrial fibrillation (star af): a randomized, multicentre, international trial. Eur Heart J. (2010) 31:1344–56. 10.1093/eurheartj/ehq041 20215126 PMC2878965

[4] Verma A Sanders P Macle L Deisenhofer I Morillo CA Chen J Substrate and trigger ablation for reduction of atrial fibrillation trial-part ii (star af ii): design and rationale. Am Heart J. (2012) 164:1–6 e6. 10.1016/j.ahj.2012.04.002 22795275

[5] Jadidi AS Duncan E Miyazaki S Lellouche N Shah AJ Forclaz A Functional nature of electrogram fractionation demonstrated by left atrial high-density mapping. Circ Arrhythm Electrophysiol. (2012) 5:32–42. 10.1161/CIRCEP.111.964197 22215849 PMC3423961

[6] Rostock T Rotter M Sanders P Takahashi Y Jaïs P Hocini M High-density activation mapping of fractionated electrograms in the atria of patients with paroxysmal atrial fibrillation. Heart Rhythm. (2006) 3:27–34. 10.1016/j.hrthm.2005.09.019 16399048

[7] Haïssaguerre M Hocini M Sanders P Takahashi Y Rotter M Sacher F Localized sources maintaining atrial fibrillation organized by prior ablation. Circulation. (2006) 113:616–25. 10.1161/CIRCULATIONAHA.105.546648 16461833

[8] Takahashi Y O'Neill MD Hocini M Dubois R Matsuo S Knecht S Characterization of electrograms associated with termination of chronic atrial fibrillation by catheter ablation. J Am Coll Cardiol. (2008) 51:1003–10. 10.1016/j.jacc.2007.10.056 18325439

[9] Seitz J Bars C Theodore G Beurtheret S Lellouche N Bremondy M Af ablation guided by spatiotemporal electrogram dispersion without pulmonary vein isolation: a wholly patient-tailored approach. J Am Coll Cardiol. (2017) 69:303–21. 10.1016/j.jacc.2016.10.065 28104073 PMC5568427

[10] Aksu T Guler TE Yalin K Oto A. Unanswered questions in complex fractionated atrial electrogram ablation. Pacing Clin Electrophysiol. (2016) 39:1269–78. 10.1111/pace.12944 27566694

[11] Lin YJ Tsao HM Chang SL Lo LW Hu YF Chang CJ Role of high dominant frequency sites in nonparoxysmal atrial fibrillation patients: insights from high-density frequency and fractionation mapping. Heart Rhythm. (2010) 7:1255–62. 10.1016/j.hrthm.2010.06.019 20558322

[12] Bollmann A Husser D Mainardi L Lombardi F Langley P Murray A Analysis of surface electrocardiograms in atrial fibrillation: techniques, research, and clinical applications. Europace. (2006) 8:911–26. 10.1093/europace/eul113 17043067

[13] Rieta JJ Castells F Sánchez C Zarzoso V Millet J. Atrial activity extraction for atrial fibrillation analysis using blind source separation. IEEE Trans Biomed Eng. (2004) 51:1176–86. 10.1109/TBME.2004.827272 15248534

[14] Zarzoso V Comon P. Robust independent component analysis by iterative maximization of the kurtosis contrast with algebraic optimal step size. IEEE Trans Neural Netw. (2010) 21:248–61. 10.1109/TNN.2009.2035920 20028621

[15] Pokushalov E Romanov A Corbucci G Artyomenko S Turov A Shirokova N Use of an implantable monitor to detect arrhythmia recurrences and select patients for early repeat catheter ablation for atrial fibrillation: a pilot study. Circ Arrhythm Electrophysiol. (2011) 4:823–31. 10.1161/CIRCEP.111.964809 21930653

[16] Pokushalov E Romanov A Corbucci G Bairamova S Losik D Turov A Does atrial fibrillation burden measured by continuous monitoring during the blanking period predict the response to ablation at 12-month follow-up? Heart Rhythm. (2012) 9:1375–9. 10.1016/j.hrthm.2012.03.047 22449740

[17] Andrade JG Khairy P Macle L Packer D Lehmann JW Holcomb RG Incidence and significance of early recurrences of atrial fibrillation after cryoballoon ablation: insights from the multicenter stop af trial. Circ Arrhythm Electrophysiol. (2014) 7(1):69–75. 10.1161/circep.113.000586 24446022

[18] Jadidi AS Cochet H Shah AJ Kim SJ Duncan E Miyazaki S Inverse relationship between fractionated electrograms and atrial fibrosis in persistent atrial fibrillation: combined magnetic resonance imaging and high-density mapping. J Am Coll Cardiol. (2013) 62:802–12. 10.1016/j.jacc.2013.03.081 23727084

[19] Morgan R Colman MA Chubb H Seemann G Aslanidi OV. Slow conduction in the border zones of patchy fibrosis stabilizes the drivers for atrial fibrillation: insights from multi-scale human atrial modeling. Front Physiol. (2016) 7:474. 10.3389/fphys.2016.00474 27826248 PMC5079097

[20] Deng D Murphy MJ Hakim JB Franceschi WH Zahid S Pashakhanloo F Sensitivity of reentrant driver localization to electrophysiological parameter variability in image-based computational models of persistent atrial fibrillation sustained by a fibrotic substrate. Chaos. (2017) 27:093932. 10.1063/1.5003340 28964164 PMC5605332

[21] Cochet H Dubois R Yamashita S Al Jefairi N Berte B Sellal JM Relationship between fibrosis detected on late gadolinium-enhanced cardiac magnetic resonance and re-entrant activity assessed with electrocardiographic imaging in human persistent atrial fibrillation. JACC Clin Electrophysiol. (2018) 4:17–29. 10.1016/j.jacep.2017.07.019 29479568 PMC5824731

[22] Narayan SM Krummen DE Shivkumar K Clopton P Rappel WJ Miller JM. Treatment of atrial fibrillation by the ablation of localized sources: confirm (conventional ablation for atrial fibrillation with or without focal impulse and rotor modulation) trial. J Am Coll Cardiol. (2012) 60:628–36. 10.1016/j.jacc.2012.05.022 22818076 PMC3416917

[23] Sanders P Berenfeld O Hocini M Jaïs P Vaidyanathan R Hsu LF Spectral analysis identifies sites of high-frequency activity maintaining atrial fibrillation in humans. Circulation. (2005) 112:789–97. 10.1161/CIRCULATIONAHA.104.517011 16061740

[24] Atienza F Almendral J Jalife J Zlochiver S Ploutz-Snyder R Torrecilla EG Real-time dominant frequency mapping and ablation of dominant frequency sites in atrial fibrillation with left-to-right frequency gradients predicts long-term maintenance of sinus rhythm. Heart Rhythm. (2009) 6:33–40. 10.1016/j.hrthm.2008.10.024 19121797 PMC2867332

[25] Guillem MS Climent AM Rodrigo M Fernández-Avilés F Atienza F Berenfeld O. Presence and stability of rotors in atrial fibrillation: evidence and therapeutic implications. Cardiovasc Res. (2016) 109:480–92. 10.1093/cvr/cvw011 26786157 PMC4777913

[26] Okumura Y Watanabe I Kofune M Nagashima K Sonoda K Mano H Characteristics and distribution of complex fractionated atrial electrograms and the dominant frequency during atrial fibrillation: relationship to the response and outcome of circumferential pulmonary vein isolation. J Interv Card Electrophysiol. (2012) 34:267–75. 10.1007/s10840-011-9637-2 22205497

[27] Yoshida K Chugh A Good E Crawford T Myles J Veerareddy S A critical decrease in dominant frequency and clinical outcome after catheter ablation of persistent atrial fibrillation. Heart Rhythm. (2010) 7:295–302. 10.1016/j.hrthm.2009.11.024 20117058

[28] Atienza F Almendral J Ormaetxe JM Moya A Martínez-Alday JD Hernández-Madrid A Comparison of radiofrequency catheter ablation of drivers and circumferential pulmonary vein isolation in atrial fibrillation: a noninferiority randomized multicenter radar-af trial. J Am Coll Cardiol. (2014) 64:2455–67. 10.1016/j.jacc.2014.09.053 25500229

[29] Verma A Lakkireddy D Wulffhart Z Pillarisetti J Farina D Beardsall M Relationship between complex fractionated electrograms (cfe) and dominant frequency (df) sites and prospective assessment of adding df-guided ablation to pulmonary vein isolation in persistent atrial fibrillation (af). J Cardiovasc Electrophysiol. (2011) 22:1309–16. 10.1111/j.1540-8167.2011.02128.x 21736659

[30] Fischer G Stühlinger M Nowak CN Wieser L Tilg B Hintringer F. On computing dominant frequency from bipolar intracardiac electrograms. IEEE Trans Biomed Eng. (2007) 54:165–9. 10.1109/TBME.2006.883739 17260870

[31] Baykaner T Lalani GG Schricker A Krummen DE Narayan SM. Mapping and ablating stable sources for atrial fibrillation: summary of the literature on focal impulse and rotor modulation (firm). J Interv Card Electrophysiol. (2014) 40(3):237–44. 10.1007/s10840-014-9889-8 24647673

